# Effect of Fe–O ReaxFF on Liquid Iron Oxide Properties Derived from Reactive Molecular Dynamics

**DOI:** 10.1021/acs.jpca.3c06646

**Published:** 2023-11-20

**Authors:** Leon C. Thijs, Efstratios M. Kritikos, Andrea Giusti, Marie-Aline van Ende, Adri C. T. van Duin, XiaoCheng Mi

**Affiliations:** † Department of Mechanical Engineering, Eindhoven University of Technology, P.O. Box 513, Eindhoven 5600 MB, Netherlands; ‡ Department of Mechanical Engineering, Imperial College London, London SW7 2AZ, U.K.; § Department of Materials Science and Engineering, Research Institute of Advanced Materials, Seoul National University, Seoul 08826, South Korea; ∥ Department of Mechanical Engineering, The Pennsylvania State University, University Park, Pennsylvania 16802, United States; ⊥ Eindhoven Institute of Renewable Energy Systems, Eindhoven University of Technology, Eindhoven 5600 MB, Netherlands

## Abstract

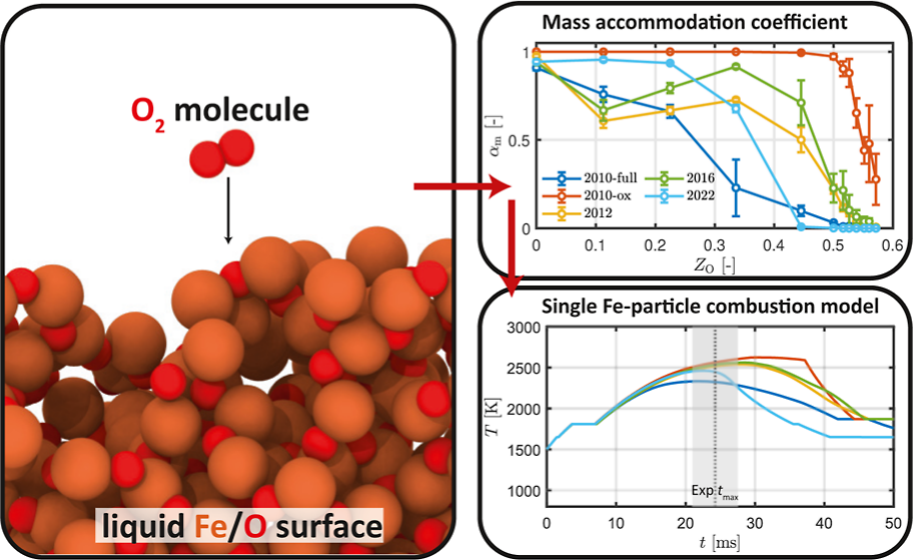

As iron powder nowadays attracts research attention as a carbon-free, circular energy carrier, molecular dynamics (MD) simulations can be used to better understand the mechanisms of liquid iron oxidation at elevated temperatures. However, prudence must be practiced in the selection of a reactive force field. This work investigates the influence of currently available reactive force fields (ReaxFFs) on a number of properties of the liquid iron–oxygen (Fe–O) system derived (or resulting) from MD simulations. Liquid Fe–O systems are considered over a range of oxidation degrees *Z*_O_, which represents the molar ratio of O/(O + Fe), with 0 < *Z*_O_ < 0.6 and at a constant temperature of 2000 K, which is representative of the combustion temperature of micrometric iron particles burning in air. The investigated properties include the minimum energy path, system structure, (im)miscibility, transport properties, and the mass and thermal accommodation coefficients. The properties are compared to experimental values and thermodynamic calculation results if available. Results show that there are significant differences in the properties obtained with MD using the various ReaxFF parameter sets. Based on the available experimental data and equilibrium calculation results, an improved ReaxFF is required to better capture the properties of a liquid Fe–O system.

## 1 Introduction

Nowadays, the scientific community has shifted its interest in alternative fuels to decarbonize the transportation and energy sectors. Over the past years, the interest in using metal fuels, especially iron powder, as a circular carrier of renewable energy has drastically increased. Iron powder is considered as a promising carbon-free, recyclable, compact, and cheap energy carrier.^1^

To design and improve real-world iron-fuel burners, an in-depth understanding of the fundamentals underlying the combustion of single iron particles is required. In the past few years, the number of more detailed experimental^2−10^ and theoretical studies^11−18^ regarding the combustion of single iron particles has increased drastically. In this early research on iron particle combustion, a good agreement between experiments and theoretical models for low gas temperature (300 K) and low oxygen concentration cases (up to *X*_O2_ = 0.21) was obtained. However, the theoretical models were not able to capture two distinct phenomena. First, the model overestimates the maximum particle temperature at elevated oxygen concentrations.^14,17^ Second, the model is not able to reproduce the positive correlation between the particle size and maximum particle temperature,^14^ as observed by Ning et al.^3^ Despite the good agreement between experiments and theoretical models for low gas temperature and low oxygen concentration cases, fundamental knowledge of the complex physical and chemical mechanisms in iron particle combustion is still very limited.

For iron particle combustion, it has been hypothesized that the oxidation rate of an iron droplet is the result of an interplay among three mechanisms: (1) external diffusion of O_2_ from the ambient gas to particle surface, (2) surface chemisorption of O_2_, and (3) internal transport of Fe and O atoms.^19^ To describe the limiting process (2), the mass accommodation coefficient (MAC) between Fe_*x*_O_*y*_ and O_2_ must be known. For describing the limiting process (3), more information about the particle internal structure as well as the transport properties, diffusivity and viscosity, of the liquid Fe–O system must be known.

The internal structure of the particle in the liquid phase is rather a complex and not well-understood process. Muller et al.^20^ investigated the laser ignition of pure (purity, 99.99%) iron rods. They observed that liquid iron (L1) and liquid iron oxide (L2) phases can be either distinct and immiscible or mixed together. These experimental observations seem to indicate a complex oxidation process in the liquid, for instance, with L1 also being present at the particle surface and, under certain circumstances, miscibility between phases L1 and L2. In addition to the rather complex phenomena occurring during liquid-phase combustion, physical properties of the liquid Fe–O system are not always well-known at high temperatures (above 2000 K) and atmospheric pressure. For example, transport properties, such as viscosity and diffusion coefficients of Fe and O ions, have not been extensively studied under these conditions but are of importance to model the convective flow and the internal structure in an oxidizing liquid iron droplet. The properties of the liquid Fe–O system are extensively investigated by Earth and planetary scientists because of the abundance of liquid iron and its oxides in Earth’s outer core.^21−25^ However, the required properties are typically determined at elevated pressures of around 100–350 GPa, which differ significantly from atmospheric conditions.

In order to advance the theoretical models, a more comprehensive understanding of the underlying physical and chemical mechanisms inherent in iron particle combustion is needed. However, since the properties like MAC, L1–L2 (im)miscibility, diffusivity, and viscosity of liquid Fe–O system do not always exist in the literature, or the experimental measurements are difficult to perform, molecular dynamics simulations can be performed to gain insights into the phenomena occurring during the liquid phase combustion and to determine the physical properties of a liquid Fe–O system at atmospheric pressure.

In this context, reactive molecular dynamics (MD) simulations enabled by reactive force fields (ReaxFFs)^26^ have proven their ability to provide fundamental insights into the oxidation of gas, liquid, and solid fuels, predicting material properties and other physicochemical processes.^27^ For the specific case of iron, (reactive) molecular dynamics simulations have been used for various applications: to determine the material properties of pure liquid iron,^28^ the oxidation of nanoparticles and surfaces with oxygen,^29−31^ the oxidation of Fe with CO_2_^32,33^ and H_2_O,^34^ and also for the oxidation of alloys.^35^ In the study of Thijs et al.^14^ reactive MD simulations were used to investigate the thermal and mass accommodation coefficients (TAC and MAC, respectively) for the combination of high-temperature iron(-oxide) and air. It should be noted that these (reactive) MD simulation results are to some extent limited by the accuracy and availability of the (reactive) force fields. Therefore, to build confidence in MD results, it is important to assess to what extent an MD-ReaxFF simulation can reproduce a wide range of physicochemical properties of the liquid Fe–O system.

The development of a ReaxFF with adequate accuracy under certain conditions (in terms of temperature and pressure) is a complex task. In ReaxFF, multiple terms contribute to the total energy, while for nonreactive force fields, typically a single interaction type determines the energy contributions. ReaxFF parameter sets are often calibrated for a specific application, implying that applying ReaxFF to a slightly different application outside the calibrated range can lead to inaccurate results. Therefore, prudence must be practiced in the selection of force fields.

The goal of this study is to investigate and assess the capability of different ReaxFF parameter sets available for the Fe–O system to predict the following liquid Fe–O properties that are important when considering the combustion of iron particles:

1. Minimum energy path (MEP): The processes of oxygen adsorption on an iron surface and oxygen diffusion within an iron slab are investigated through nudge elastic band (NEB) computations.^36^ The results are compared to relevant density functional theory (DFT) literature values.
2. System structure: An assessment of density in a liquid Fe–O system is conducted, and the derived data are compared with the existing literature data and results from thermodynamic calculations. Furthermore, the obtained radial distribution functions (RDFs) are investigated to gain more insights into the predicted surface structures.
3. (Im)miscibility: According to the Fe–O phase diagram, there is a miscibility gap where liquid iron and liquid iron oxide do not mix.^37^ In the context of combustion involving micron-sized iron particles, a crucial aspect is gaining insights into the potential mixing of liquid Fe and liquid FeO within the high-temperature and dynamically changing environments. Therefore, the demixing behavior in an L1–L2 melt predicted by the different ReaxFF parameter sets is investigated by examining the phase separation and enthalpy of mixing. The latter parameter will be compared to thermodynamic calculations. For the miscible region of the phase diagram, the coordination numbers predicted by the different ReaxFF parameter sets will be compared to the values provided by Shi et al.^38^ They measured the Fe–O coordination numbers of molten iron oxides and found that the Fe–O coordination number in the region of liquid FeO to liquid Fe_2_O_3_ ranges between 4.5 and 5.
4. Transport properties: To model the internal structure of a liquid iron oxide droplet during combustion, the diffusion coefficients of Fe and O as well as the viscosity are needed to model the internal diffusion and convection. The transport properties, viscosity and diffusion coefficients of Fe and O ions, will be compared to the available literature data and results from thermodynamic calculations.
5. Mass and thermal accommodation coefficients: This investigation involves the determination of MAC and TAC between Fe_*y*_O_*x*_ and O_2_. Both MAC and TAC are critical parameters that directly impact the oxidation efficiency and heat transfer efficiency during iron particle combustion, respectively. The impact of MAC and TAC values derived from different reactive force fields is assessed using the single iron particle model outlined by Thijs et al.^14^

The paper is organized as follows. Section 2 describes the research strategy, the methodology used for the molecular dynamics simulations, and the methodology for the thermodynamic calculations. Then, the results are presented in Section 3. Conclusions and recommendations are given in Section 4.

## 2 Methods

### 2.1 Research Strategy

To evaluate the performance of various ReaxFF parameter sets applicable to the Fe–O system, a comprehensive assessment involves investigating the five key properties listed in Section 1 through reactive molecular dynamics simulations. The study of these five key properties may be extended to subparameters to provide additional support to the findings. The simulations performed in this study employ four distinct setups: NEB1, NEB2, SysPer, and SurfVac, which will be elaborated upon later. Whenever feasible, the properties obtained from the MD simulations are compared to the values derived from DFT, experimental measurements, or thermodynamic calculations if possible. An overview of which specific MD setup is utilized for each property and the corresponding benchmark values for comparison is summarized in Table 1. An assessment will be provided categorizing the performance of different ReaxFF parameter sets as either good, moderate, or bad, in relation to the available benchmark values. Note that the terms “bad,” “moderate”, and “good” are used to describe how well certain ReaxFF parameter sets predict certain properties compared to others that may not predict those properties very accurately. Hence, the rationale behind the categorization may vary depending on the specific property in question.

**Table 1:** Overview of the MD Setup Used for Deriving the MD Properties and the Corresponding Source for the Benchmark Values

MD setup	properties	benchmark values
NEB1	1. minimum energy path	
	a. oxygen adsorption	DFT^39−41^
NEB2	1. minimum energy path	
	b. oxygen diffusion	DFT^42−45^
SysPer	2. system structure	
	a. density	experiments,^46,47^ FactSage^48^
	b. RDF	
	3. (im)miscibility	
	a. enthalpy of mixing	FactSage^48^
	b. immiscible phase: confirmation of mixing	
	c. immiscible phase: L2 composition	FactSage^48^
	d. miscible phase: coordination numbers	experiments^38^
	4. transport properties	
	a. diffusion coefficients	experiments^49−51^
	b. viscosity	experiments,^52^ FactSage^48^
SurfVac	5. mass and thermal accommodation coefficients	
	a. impact on burn time of single iron particle combustion	experiments^2^

### 2.2 Reactive Molecular Dynamics

Reactive MD uses reactive force fields to accurately describe bond formation and breaking. ReaxFF^26^ is a bond order potential that describes the total energy of the system as
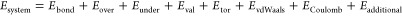
1where *E*_bond_ is the bond formation/breaking energy, *E*_over_ and *E*_under_ are the over- and undercoordination energy penalties, *E*_val_ and *E*_tor_ are, respectively, the valence and torsion angle energies, *E*_vdWaals_ and *E*_Coulomb_ are the nonbonded van der Waals and Coulomb long-range interactions, and *E*_additional_ are additional correction terms. The atomic charges are computed at every time step using the charge equilibration method. All simulations are performed using a large-scale atomic/molecular massively parallel simulator (LAMMPS).^53^

Over the past few years, several improved force fields for iron have been reported.^54−61^ Here, the impact of six different reactive force fields on the prediction of liquid iron oxide properties will be investigated, including those of Aryanpour et al.^54^ (ReaxFF2010-ox and ReaxFF2010-full), Zou and van Duin^55^ (ReaxFF2012), Shin et al.^56^ (ReaxFF2015), Islam et al.^57^ (ReaxFF2016), and Huang et al.^58^ (ReaxFF2022). Each ReaxFF parameter set was trained and used for a specific application:

- The ReaxFF reactive force field, developed by Aryanpour et al.,^54^ is widely used to simulate the oxidation of Fe with either water or oxygen. They developed two ReaxFF parameter sets focusing on iron oxides and iron oxyhydroxides, denoted in this study as ReaxFF2010-ox and ReaxFF2010-full, respectively.
- Zou and van Duin^55,62^ (ReaxFF2012) developed a ReaxFF parameter set specifically designed to investigate the Fischer–Tropsch (FT) synthesis process. FT synthesis involves the conversion of hydrogen and carbon monoxide into various hydrocarbons, often catalyzed by metals such as iron. These reactions typically occur within the temperature range of 470–620 K.^55^ Zou and van Duin^55,62^ used the O/H/Fe interactions proposed by Aryanpour et al.^54^ to optimize their ReaxFF parameter set.
- Shin et al.^56^ (ReaxFF2015) developed a ReaxFF force field to investigate the Cr oxide-catalyzed oxidation reaction of butane at a high temperature of 1600 K. This study considered the presence of iron pyrite (FeS_2_), which can accelerate the complete oxidation of butane.
- Islam et al.^57^ (ReaxFF2016) developed their ReaxFF parameter sets to study the interaction of hydrogen with pure and defective ferrite–cementite interfaces. They merged the ReaxFF force field that was developed for the FT synthesis (Fe–C–H system)^55,62^ with a ReaxFF carbon parameter set^63^ and subsequently optimized the parameters.
- More recently, Huang et al.^58^ (ReaxFF2022) investigated the corrosion mechanism of solid iron surfaces at 973, 1173, and 1373 K with water. Their research identified limitations in the ReaxFF parameter set proposed by Aryanpour et al.,^54^ particularly in accurately describing the diffusion behaviors of hydrogen and oxygen within iron. Therefore, they improved the initial parameters of Aryanpour et al.^54^ to optimize the Fe–Fe, Fe–O, and Fe–H parameter sets.

Among all of these ReaxFF Fe/O descriptions, the ReaxFF2015 potential is primarily oriented toward bulk oxides, with its training set encompassing heats of formation and equations of state for Fe, FeO, Fe_3_O_4_, and Fe_2_O_3_. In contrast, the ReaxFF2012 and ReaxFF2016 force fields place greater emphasis on Fe surface chemistry, whereas the ReaxFF2010 potential is more centered on iron oxyhydroxide/iron oxide conversion reactions. It is worth noting that the ReaxFF parameter sets discussed above are predominantly designed for broader applications beyond Fe–O interactions, with a primary emphasis on solid-state phenomena.

### 2.3 MD Simulation Details

Four distinct setups will be used to investigate five key parameters: NEB1, NEB2, SysPer, and SurfVac. The different setups are detailed below.

#### 2.3.1 NEB1 and NEB2

NEB computations are performed to predict the oxygen adsorption on an iron surface and oxygen diffusion in an iron slab. These simulations employ two distinct MD setups, denoted as NEB1 and NEB2, to address the aspects of adsorption and diffusion.

For the initial state of the adsorption reaction, the O atom is placed at a distance of around 5.5° A from an Fe(100) surface consisting of 432 Fe atoms. This system is denoted as NEB1. The final state was created by placing the O atom at the hollow site of the iron surface, which is the most energetically favorable one.^40^ After the minimization of the final state, the position of the O atom varies.

**Figure 1:**
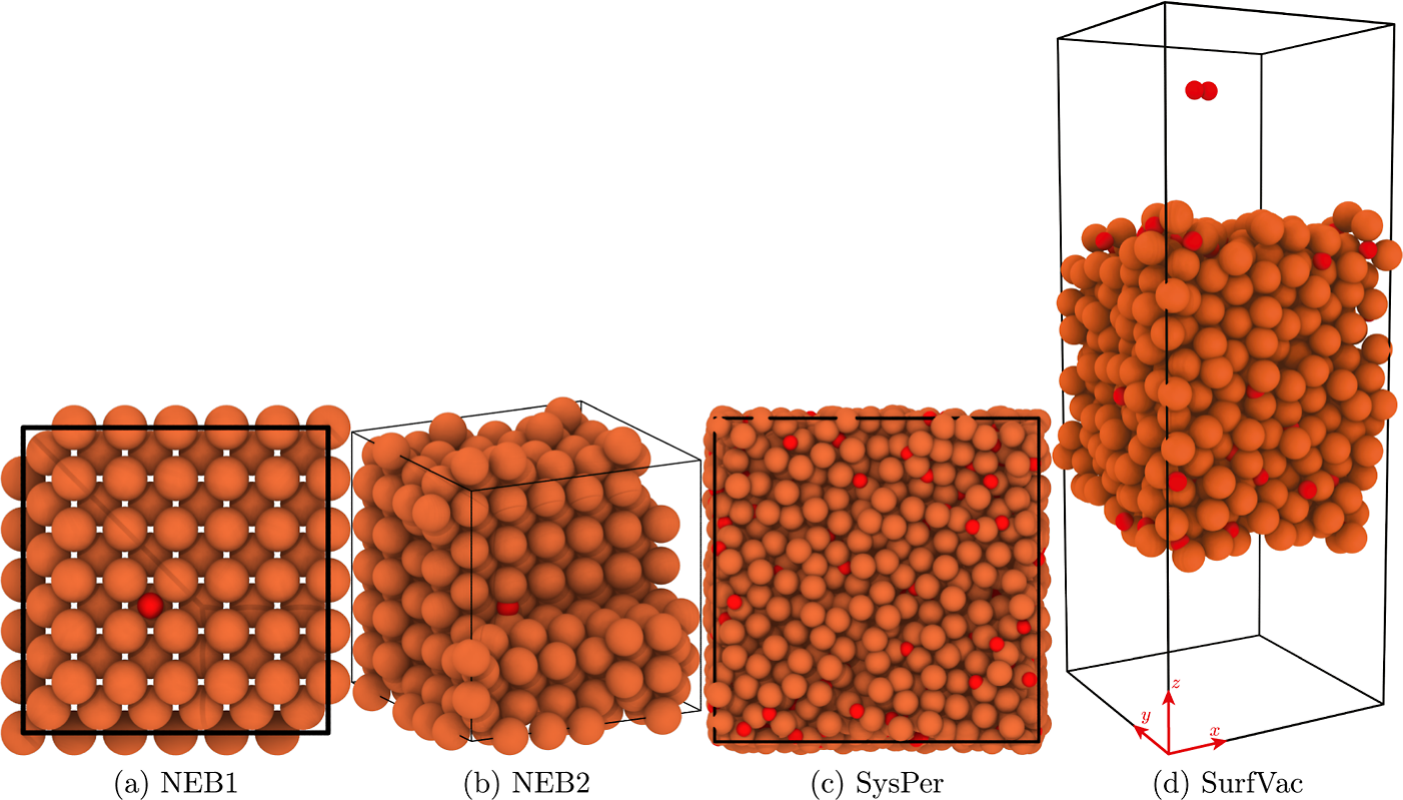
Final configurations used to determine the MEP properties (NEB1 and NEB2), system structure, (im)miscibility and transport properties (SysPer), and MAC and TAC (SurfVac). For NEB2, a part of the domain is not shown to visualize the position of the oxygen atom. The periodic boundaries are indicated with black lines. CPK coloring is used to distinguish different chemical elements, where an oxygen atom is depicted in red and an iron atom in brown.

For investigating oxygen diffusion in iron, the initial and final states of O diffusion in iron correspond to two adjacent octahedral interstitial sites. This system is denoted as NEB2. Figure 1a,b show the final configurations used for the NEB1 and NEB2 setups. Before performing NEB simulations, the iron slab undergoes an energy minimization process following the conjugate gradient method.

#### 2.3.2 SysPer Conditions

To investigate the key parameters describing the system structure, (im)miscibility, and transport properties, a system with periodic (SysPer) boundary conditions in all three directions is used.

Different liquid iron oxide structures at a constant temperature of 2000 K, which is representative of the combustion temperature of micrometric iron particles burning in air, are generated to investigate the impact of the ReaxFF parameter sets on different oxidation stages. The oxidation stage is denoted by the elemental mole fraction of oxygen in the particle and can be calculated as
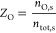
2with *n*_O,s_ being the number of oxygen atoms and *n*_tot,s_ the total number of atoms in the complete domain.

In the case of *Z*_O_ < 0.5, before applying the thermostats, an FeO lattice is deposited in a specific ratio on top of a BCC lattice of Fe atoms. If *Z*_O_ > 0.5, an Fe_3_O_4_ lattice is deposited on top of an FeO lattice. A vacuum on top and bottom of the domain is added, and an annealing process is employed for all the surfaces to eliminate the initial crystalline structure and to prepare the liquid-phase structure of *T*_p_ = 2000 K. The surfaces are heated using the canonical (*NVT*) ensemble for 30 ps to 2800 K, equilibrated for 30 ps at the same temperature, and then gradually cooled down to the target temperature of 2000 K within 30 ps. The heated surfaces are then allowed to continue in the *NVT* ensemble for 5 ps, after which the domain is adjusted to the volume of the liquid iron oxide. Then, the production simulation starts in the microcanonical (*NVE*) ensemble and lasts for 0.1 ns. To keep a constant temperature in the *NVT* ensemble simulations, the Nose–Hoover thermostat is applied on the translational degrees of freedom of the atoms with a temperature damping period of 10 fs. A time step of 0.1 fs is used, which is recommended for reactive MD simulations at high temperatures.^64^Figure 1c shows the initial configuration used for the SysPer domain.

#### 2.3.3 SurfVac

To investigate the MACs between Fe_*x*_O_*y*_ and O_2_, the same configuration as in ref (14) is used. Therefore, above and below the surface, a vacuum (SurfVac) is added to simulate an incoming oxygen molecule. After the surface realization, an O_2_ molecule is located around 10 Å above the surface, beyond the range of the potential well. Three different surfaces, each with different initial velocities, are generated to obtain a statistically meaningful set of data for the MACs. Then, incident gas molecules are introduced, with their velocities sampled from the Maxwell–Boltzmann distribution. 500 cases per warmed surface are sampled, which result in 1500 data points per configuration. A time step of 0.1 fs is used. Figure 1d shows the initial configuration used for the interaction between Fe_*x*_O_*y*_ and O_2_.

### 2.4 Thermodynamic Calculations

Thermodynamic calculations were performed by using the FactSage thermochemical software (version 8.2)^48^ and the FTsulf database. The thermodynamic description of the Fe–O system in FactSage is based on the critical evaluation and optimization of the Fe–O system by Hidayat et al.^37^ In their optimization, the Gibbs energy of the liquid solution was modeled using the modified quasichemical model (MQM),^65^ which considers the short-range ordering between components in the liquid phase. Moreover, as information on the structure of the melt can be directly obtained from the thermodynamic description of the melt using the MQM implemented in FactSage, structural viscosity^66,67^ and molar volume^68^ models were successfully developed for multicomponent oxide melts. In this study, these models were used to calculate the viscosity and density of liquid FeO.

## 3 Results and Discussion

### 3.1 Minimum Energy Path

The ReaxFF parameter sets investigated in this work are often calibrated for broader applications than just the Fe–O interaction, for example, by including C or H interactions in the reactive force field. Therefore, when the focus is on Fe–O systems only, the accuracy of the prediction of the interactions between iron and oxygen must be evaluated. The ability of various ReaxFF force fields to predict oxygen adsorption on an iron surface and oxygen diffusion in an iron slab is investigated using NEB computations^36^ with the climbing image (CI)^69^ method.

The NEB method can be used to find a reaction path and to identify the transition state between a reactant and the product configuration. For the NEB calculation, an initial approximation of the reaction path is established, wherein a series of images are generated through linear interpolation spanning between the initial and final systems. The actual reaction path is then determined by conducting a simultaneous optimization of all of these images. In the NEB method, the images are not independent from each other: the force acting on each image is dependent on the images adjacent to it. Forces parallel to the reaction path are eliminated during each optimization step, and a spring force is introduced. This force aims to keep each image equidistant from its neighbors. This mechanism prevents images from sliding toward the initial or final reaction states and ensures that they are distributed uniformly along the reaction path. A CI algorithm is used throughout the NEB path optimization to guide the highest-energy image toward the transition state. In the specific implementation of this study, a total of 20 images is used to obtain a detailed description of the MEP. The spring force is set to be 1 kcal/(mol Å).

#### 3.1.1 Oxygen Adsorption

**Figure 2:**
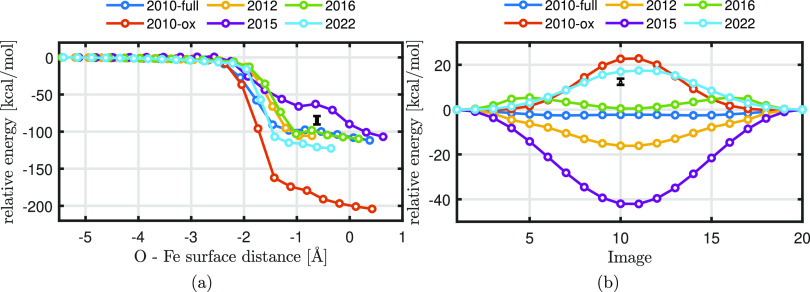
NEB results for the (a) adsorption reaction of an oxygen atom on the hollow site of an Fe(100) surface and (b) diffusion of an oxygen atom in iron. The initial and final states of O diffusion in iron correspond to two adjacent octahedral interstitial sites. The black error bar indicates the mean and standard deviation of the DFT calculation results for adsorption^39−41^ and diffusion.^42−45^

Figure 2a shows the NEB results for the adsorption reaction of an oxygen atom on the hollow site of an Fe(100) surface. The total energy obtained with the NEB computation is taken relative to the total energy of the initial configuration, while the Fe–O distance *z* is the position of oxygen with respect to the positions of the outermost iron atoms. The adsorption energy *E*_ad_ and the adsorption distance *z*_ad_ are the relative energy and the Fe–O distance at the final position, respectively. The result obtained with the different ReaxFF parameter sets are compared to the DFT calculations available in the literature.^39−41^Table 2 lists the ReaxFF- and DFT-predicted adsorption energies *E*_ad_ and the normal distance *z* obtained at the final position. These results reveal that ReaxFF2010-ox significantly overestimates the change in potential energy. This overestimation of the change in potential energy indicates that ReaxFF2010-ox predicts a too strong interaction between the O atom and the Fe surface. All other ReaxFF parameter sets overestimate the adsorption energy by at least 20 kcal/mol. The final position of the oxygen atom with respect to the Fe surface differs for the different ReaxFFs, resulting in positions either below or above the outermost iron atoms. ReaxFF2015 and ReaxFF2016 do predict a local minimum in the potential energy near the −1 Å distance which implies an energetically favorable adsorption location. Such local minima are not particularly evident for the other ReaxFF parameter sets. Since ReaxFF2010-ox overestimates *E*_ad_ by more than 100%, we categorize this ReaxFF as “bad” for predicting absorption reactions. ReaxFF2012 is categorized as “good” because it predicts *z*_ad_ within 13% and *E*_ad_ within 25%. The others are considered as “moderate” since they are not precise in predicting *z*_ad_ but can predict *E*_ad_ within at least 45%.”

**Table 2:** Adsorption Energy *E*_ed_ and Normal Distance *z*_ad_ of the Oxygen Atom to the Averaged Fe Surface at the Final Stage

case	*z*_ad_ [Å]	*E*_ad_ [kcal/mol]
DFT^39^	–0.63	–85.79
ReaxFF2010-full	+0.38	–111.80
ReaxFF2010-ox	+0.42	–204.09
ReaxFF2012	–0.71	–105.71
ReaxFF2015	+0.63	–106.91
ReaxFF2016	+0.17	–109.58
ReaxFF2022	–0.35	–122.55

#### 3.1.2 Oxygen Diffusion

Figure 2b shows the relative energy during the diffusion of an oxygen atom from one octahedral interstitial site to a neighboring site via a tetrahedral site. Based on DFT calculations, the energy barrier associated to diffusion are in the range of 11.07 and 13.84 kcal/mol,^42−45^ with the maximum value located at the tetrahedral site.^58^ ReaxFF2010-full, ReaxFF2012, and ReaxFF2015 predict a minimum energy path (MEP) with negative values, where the latter two significantly deviate from zero. This implies that the diffusion process is energetically favorable for ReaxFF2010-full, ReaxFF2012, and ReaxFF2015, while DFT simulations show otherwise. ReaxFF2016 shows an overall positive energy barrier but a decrease in energy at the tetrahedral site. Only ReaxFF2010-ox and ReaxFF2022 predict the positive MEP with the highest value at the tetrahedral location, with ReaxFF2022 being the most accurate in predicting the energy barrier. Therefore, ReaxFF2010-ox and ReaxFF2022 are the most suitable ReaxFF parameter sets to predict diffusion coefficients and are categorized as “good” for predicting oxygen diffusion, while the others are categorized as “bad”.

### 3.2 System Structure

The system structures predicted by MD are evaluated through an analysis of the density of the liquid Fe–O system. These derived data are then compared against the established literature data and outcomes from thermodynamic calculations. Moreover, the obtained RDFs are investigated to further enhance the understanding of the predicted system structures.

#### 3.2.1 Density

In the study of Thijs et al.,^14^ the effects of ReaxFF2010-full and ReaxFF2016 on the density of solid and liquid FeO and on magnetite (solid Fe_3_O_4_) were investigated. It was found that at high temperatures, the density of FeO predicted by ReaxFF-2016 agreed better with experimental results, while at solid-phase temperatures, ReaxFF-2010 agreed better with experiments, and ReaxFF2016 was not able to capture the phase change. For magnetite, the densities predicted by the two ReaxFF parameter sets were lower than the experimental results, while the density predicted by ReaxFF2010-full was in better agreement with experiments. Here, the different ReaxFF parameter sets will be assessed based on the predicted density of liquid iron oxides at 2000 K. The density is obtained before removing the vacuum and adjusting the domain to the volume of the liquid iron oxide toward the SysPer setup. The volume and subsequently the density of the liquid iron oxide system are determined by means of the alpha-shape method incorporated in the OVITO software.^70^

**Figure 3:**
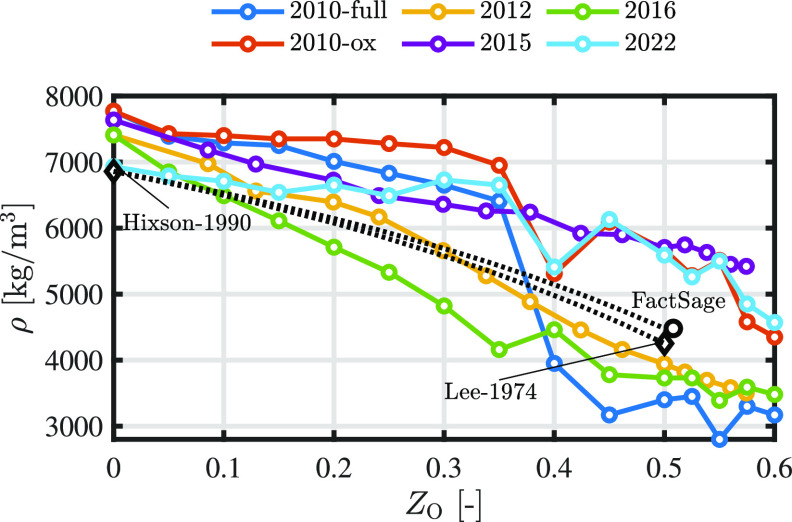
Trends in the MD density of liquid iron oxide surfaces as a function of the oxidation degree. The surface temperature is equal to 2000 K. Experimentally obtained values (black diamonds) and the density calculated with FactSage (black circle) are shown as references. The black dotted line is a linear interpolation of the experimental values based on the mass fraction of liquid Fe and liquid FeO. Note that since the mass fraction of Fe and FeO is not analogous to *Z*_O_, the line does not appear linear in this graph.

Figure 3 shows the MD-derived densities of liquid iron oxide as a function of oxidation degree at 2000 K. The experimentally obtained densities of liquid iron^46^ and the linearly extrapolated density of liquid iron oxide^47^ are added as references. The density of liquid FeO_*x*_ based on the semiempirical structural model derived from bond fractions calculated with FactSage is depicted by the black circle. The calculated density of the liquid oxide phase with a composition of 0.492 mole fraction Fe and 0.508 mole fraction O is 4480 kg/m^3^ at 2000 K. The black dotted lines are a linear interpolation of the experimental values based on the mass fraction of liquid Fe and liquid FeO. Note that since the mass fraction of Fe and FeO is not analogous to *Z*_O_, the line does not appear linear in this graph.

It is noteworthy that among the considered ReaxFF parameter sets, only ReaxFF2022 demonstrates the accurate prediction of density for pure liquid iron, while the others tend to overestimate these densities. Overall, all of the ReaxFF parameter sets predict a decreasing density with increasing *Z*_O_. Specifically, ReaxFF2015 and ReaxFF2016 exhibit a more pronounced linear decline within the range 0 < *Z*_O_ < 0.4 compared to the other models, which exhibit a notable drop around the *Z*_O_ = 0.4 point. At higher degrees of oxidation, the densities appear to stabilize at two distinct values. Specifically, ReaxFF2010-ox, ReaxFF2015, and ReaxFF2022 predict densities within the range of 4500–5500 kg/m^3^, whereas the remaining ReaxFF parameter sets predict densities ranging from 3000 to 3700 kg/m^3^. However, for the case of *Z*_O_ = 0.5, none of the ReaxFF models accurately predict the density, while ReaxFF2012 and ReaxFF2016 are the closest.

#### 3.2.2 Radial Distribution Function

**Figure 4:**
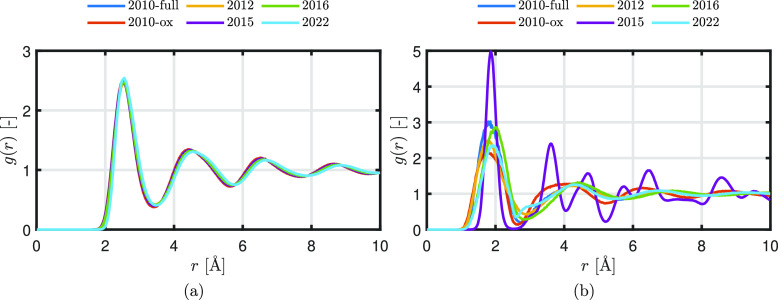
RDFs (a) *g*_FeFe_(*r*) for liquid Fe and (b) *g*_FeO_(*r*) for liquid FeO.

To gain further insights into the surface structures obtained with MD, RDFs are investigated. The RDF is a measure of the spatial distribution of atoms in a material. It provides information about the probability of finding an atom at a certain distance from the reference atom. For a liquid, the RDF shows a smooth and continuous distribution, indicating the lack of a regular ordered pattern of atoms. The RDF of a liquid usually exhibits a first peak, which corresponds to the nearest-neighbor distance, and subsequent peaks at larger distances due to the presence of higher-order neighbors. In contrast, in a solid, the RDF exhibits distinct well-defined peaks. This is because the atoms in a solid are arranged in a regular, ordered lattice structure. Figure 4 shows the RDF *g*_FeFe_(*r*) for liquid Fe and *g*_FeO_(*r*) for liquid FeO. For liquid Fe, even though the density is high and almost equal to the density of solid Fe (ρ_Fe_ = 7874 kg/m^3^), the RDF does not show a regularly ordered pattern. This implies that the structures of Fe at 2000 K predicted by all of the ReaxFF parameter sets are in a liquid phase. For FeO at 2000 K, the densities predicted by ReaxFF2010-ox, ReaxFF2015, and ReaxFF2022 are close to the density of solid FeO (ρ_FeO_ = 5745 kg/m^3^). However, from these three reactive force fields, only ReaxFF2015 shows well-defined peaks, indicating that it has a regular ordered lattice structure, while ReaxFF2010-ox and ReaxFF2012 show a typical liquid phase RDF. Therefore, we conclude that ReaxFF2015 is not able to predict the liquid iron oxide phase and categorize this ReaxFF as “bad”. Since the other ReaxFF parameter sets show a typical liquid phase and predict densities within 25%, we categorize them as “moderate”.

### 3.3 (Im)miscibility

In the context of combustion involving micrometer-sized iron particles, a crucial aspect is gaining insights into the potential mixing of liquid Fe and liquid FeO within the high-temperature and dynamically changing environments. At atmospheric pressure and at temperatures near the melting point of Fe, the solubility of oxygen in liquid iron is very low.^71^ Experimentally, it is found that the solubility of FeO in liquid Fe increases with temperature, from around 1 mol % FeO at 1811 K^72^ and 6.5 mol % FeO at 2350 K^73^ to 36 mol % FeO at 2770 K.^74^ The Fe–O equilibrium phase diagram^37^ states the existence of a miscibility gap, consisting of two immiscible liquid phases L1 (iron containing 0.013 mol % oxygen at 2000 K) and L2 (iron oxide with 0.506 mol % oxygen at 2000 K). At oxygen concentrations above 0.506 mol % and at 2000 K, the equilibrium phase diagram predicts a miscible phase. The existence of two immiscible liquids for an Fe–O system is also observed for iron combustion and iron oxide reduction experiments. Muller et al.^20^ investigated the laser ignition of pure iron rods and observed that L1 and L2 phases remain unmixed below 2350 K. Xing et al.^75^ investigated the reduction from hematite ore to metallic iron by using a high-temperature drop tube furnace. They showed that during the reduction of liquid iron oxide at 1800 K, the liquid iron and iron oxide remain separated, and the liquid iron gathers toward the center of the particle and gets enwrapped by the liquid iron oxide. In this work, the different reactive force fields are examined on the prediction of these immiscible and miscible liquid phases at 2000 K. In the context of this study, the parameters characterizing the immiscible phase are investigated within the range of 0 < *Z*_O_ < 0.5, while the miscible phase is defined as the interval 0.5 < *Z*_O_ < 0.6.

First, the immiscible phase is examined by investigating the enthalpy of mixing between liquid Fe and liquid FeO. The enthalpy of mixing is a thermodynamic concept that describes the energy changes that occur when two or more substances are combined to form a mixture. A negative enthalpy of mixing indicates that mixing is thermodynamically favorable, while a positive enthalpy implies phase separation. Then, the (im)miscibility between the two liquids is confirmed by looking at the obtained atom positions and by means of studying the probability distribution for the number of oxygen atoms neighboring an Fe atom.^71^ Then, for the ReaxFF parameter sets that predict immiscibility, the composition of the two phases is investigated to see how they compare with the L1 and L2 compositions according to the equilibrium phase diagram.

Second, the miscible liquid phase is investigated by comparing the MD-obtained Fe–O coordination number to the experiments of Shi et al.^38^

#### 3.3.1 Immiscible Phase

The enthalpy of mixing, Δ*H*_mix_, can be directly calculated from MD simulations and is defined as
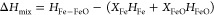
3with *H*_Fe–FeO_ the total enthalpy of the liquid Fe and liquid FeO mixture, *X*_*i*_ the molar fraction of component *i*, and *H*_*i*_ the enthalpy of pure species *i*, where *i* is either liquid Fe (*Z*_O_ = 0) or liquid FeO (*Z*_O_ = 0.5).

**Figure 5:**
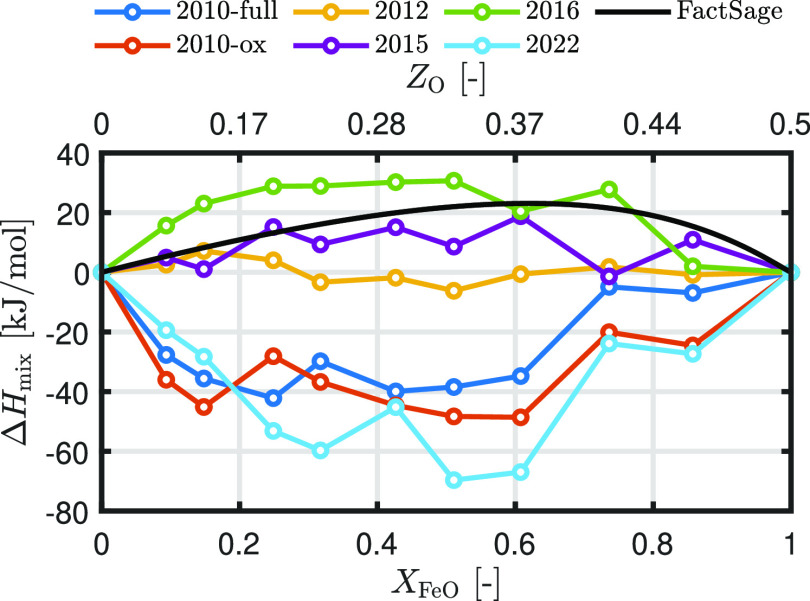
Enthalpy of mixing, Δ*H*_mix_, as a function of oxidation degree at 2000 K. The solid black line is the enthalpy of mixing calculated with FactSage.

The black solid line in Figure 5 shows the thermodynamic enthalpy of mixing between liquid Fe and liquid FeO, calculated with FactSage. It can be seen that the immiscibility is caused by a positive enthalpy of mixing between liquid Fe and liquid FeO, which indicates unfavorable mixing between the two liquids. The enthalpy of mixing increases with an increasing FeO content to a maximum of 23 kJ/mol at *X*_FeO_ = 0.6 and then decreases back to zero. The MD-derived enthalpies of mixing are compared to the data of FactSage, as shown in Figure 5. Only ReaxFF2015 and ReaxFF2016 show a relatively good agreement with the FactSage data. While the maximum enthalpy of mixing for both of these ReaxFF parameter sets is more shifted toward a smaller value of *X*_FeO_ compared to the FactSage data, it shows a positive enthalpy of mixing over the complete composition range (categorization: “good”). ReaxFF2012 shows an enthalpy of mixing which is close to zero, implying that the mixture is an ideal mixture (categorization: “moderate”). All the other ReaxFF parameter sets predict negative values for the enthalpy of mixing, implying miscibility between liquid Fe and liquid FeO (categorization: “bad”).

**Figure 6:**
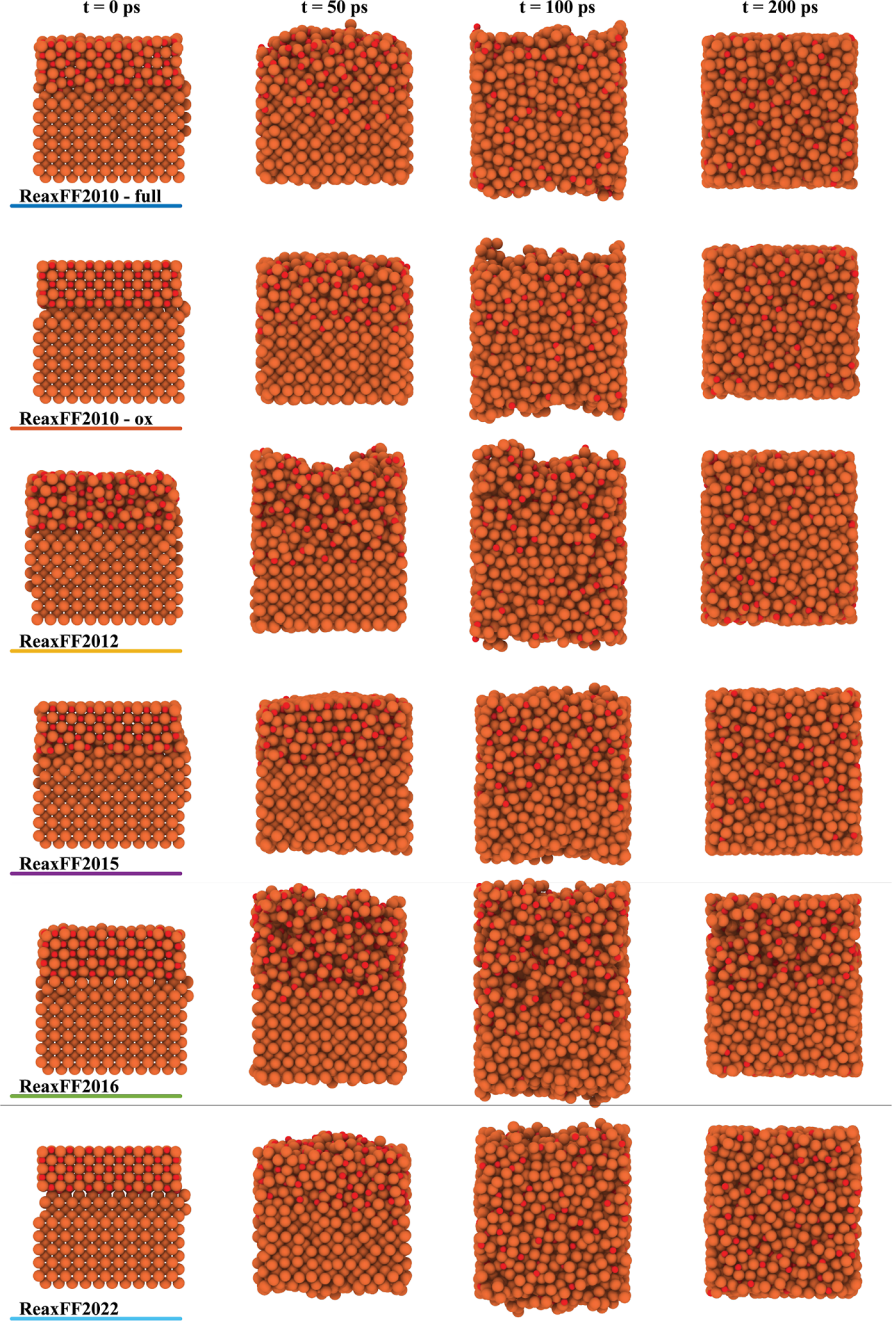
L1-L2 structures obtained at different moments in time with the different reactive force fields for *Z*_O_ = 0.2 and 2000 K. ReaxFF2010-full, ReaxFF2010-ox, and ReaxFF2022 show mixing of L1 and L2, while for ReaxFF2015 and ReaxFF2016, phase separation is observed. For ReaxFF2012, there seems a tendency toward phase separation. CPK coloring is used to distinguish different chemical elements, where an oxygen atom is depicted in red and an iron atom in brown.

To confirm the (im)miscibility between the two liquids, as predicted by the enthalpy of mixing, the obtained atom positions and the probability distribution for the number of oxygen neighbors surrounding an Fe atom are investigated.^71^Figure 6 shows the liquid Fe–liquid FeO structures obtained at different moments in time with the different reactive force fields for *Z*_O_ = 0.2 and 2000 K. ReaxFF2010-full, ReaxFF2010-ox, and ReaxFF2022 show mixing of L1 and L2, while for ReaxFF2015 and ReaxFF2016, phase separation is observed. For ReaxFF2012, there seems a tendency toward phase separation.

**Figure 7:**
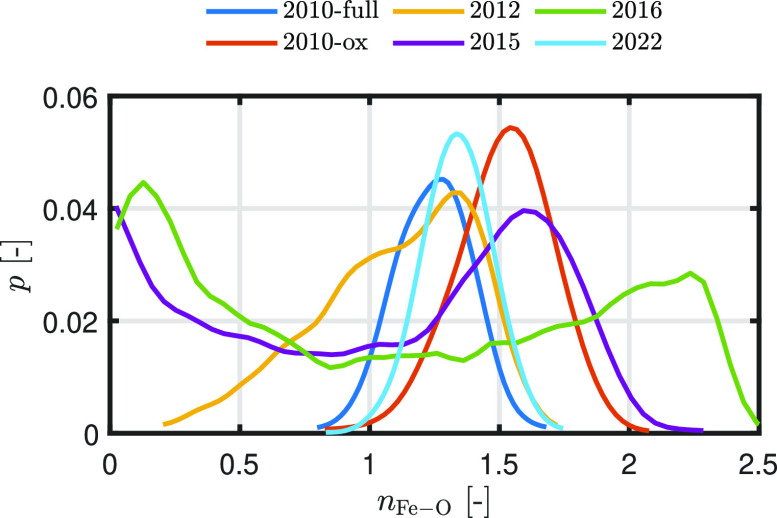
Probability of Fe–O coordination number for *Z*_O_ = 0.2 and 2000 K. The results are smoothed by means of Gaussian-weighted moving averages.

While the visual examination of the acquired surfaces provides a substantial confirmation of the prediction of two immiscible liquid phases for ReaxFF2015 and ReaxFF2016, this confirmation is less definitive in the case of ReaxFF2012. A more quantitative approach for indicating phase separation is given by the analysis of the probability distribution for the number of oxygen neighbors surrounding an Fe atom.^71^ In the case of perfect phase separation, an Fe atom in the liquid Fe phase has no oxygen neighbors, while the Fe atoms in the FeO phase have several. Figure 7 shows this probability of Fe–O coordination number for *Z*_O_ = 0.2 and 2000 K. To determine the coordination number, we used a cutoff *r* equal to the first minimum of the Fe–O RDF.^38^ For ReaxFF2015 and ReaxFF2016, two peaks can be observed, one clear peak near *n*_Fe–O_ = 0 and one peak around *n*_Fe–O_ = 1.6 and *n*_Fe–O_ = 2.2 for ReaxFF2015 and ReaxFF2016. These two peaks, with a dominant peak near *n*_Fe–O_ = 0, indicate phase separation, while this is not observed for the other ReaxFF parameter sets. This lack of peak at *n*_Fe–O_ = 0 indicates that there is no tendency toward phase separation when ReaxFF2010-full, ReaxFF2010-ox, ReaxFF2012, and ReaxFF2022 are used.

**Figure 8:**
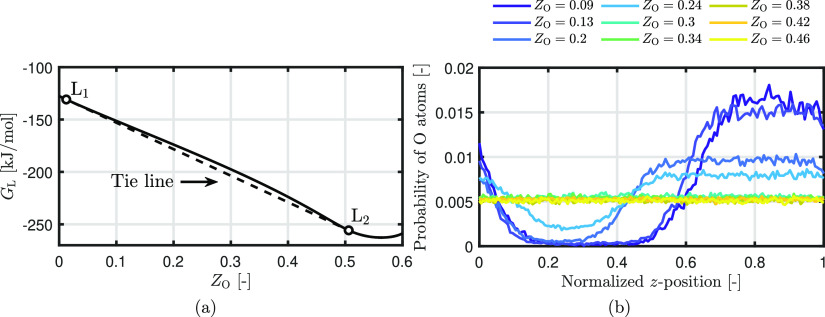
(a) Calculated liquid Gibbs energy values at miscibility gap boundaries at 2000 K. (b) Probability distribution of O atoms as a function of the dimensionless height *z* for ReaxFF2016.

It is essential to note that the equilibrium phase diagram presents specific compositions for the two immiscible liquid phases. Figure 8a shows the Gibbs energy of the Fe–O liquid phase at 2000 K calculated with FactSage. Based on the thermodynamic calculations in Figure 8a, the boundaries of the miscibility gap at a temperature of 2000 K consist of phase L1 with *Z*_O_ = 0.013 and phase L2 with *Z*_O_ = 0.506. While ReaxFF2015 and ReaxFF2016 show a reasonable agreement with the thermodynamically computed enthalpy of mixing for the liquid Fe and liquid FeO interaction, it is important to further investigate the validity of these two reactive force fields. The composition of the two phases predicted by these ReaxFF parameter sets is investigated and compared with the L1 and L2 compositions described by the equilibrium phase diagram.

To determine the composition of L2 from the MD results, first, the interface between L1 and L2 must be defined. Figure 8b shows the probability distribution of the O atoms as a function of the dimensionless height *z* for ReaxFF2016. Up to *Z*_O_ = 0.3, the phase separation is clearly visible: there are regions with a large probability (L2) and regions with a low probability of finding the O atoms (L1). Note that due to the periodic boundary conditions, these phases arise at low and high *z* values. Above *Z*_O_ = 0.3, the distribution of O atoms is uniform over the surface, even though the enthalpy of mixing is positive. This is similar for ReaxFF2015, but the threshold is at *Z*_O_ = 0.2. In our procedure, the composition of L2 is determined over the position where the probability remains constant. It is found that on average, the composition of L2 is around *Z*_O_ = 0.3 for ReaxFF16 and *Z*_O_ = 0.2 for ReaxFF2015, significantly lower than that predicted by thermodynamic calculations. This also partly explains why the phase separation is visually not visible anymore above *Z*_O_ = 0.3 and *Z*_O_ = 0.2 for ReaxFF2016 and ReaxFF2015. Since the overall oxidation degree is equal to the L2 composition, as predicted by the ReaxFF parameter sets, no phase separation occurs. Therefore, we categorize these ReaxFF parameter sets as “bad” in predicting the L2 composition, while for the other ReaxFF parameter sets, it is not possible to predict this property.

#### 3.3.2 Miscible Phase

The thermophysical and thermodynamic properties of a liquid iron oxide structure are affected by its atomic structural arrangement. Therefore, it is important that ReaxFF can accurately predict this atomic structural arrangement. Shi et al.^38^ used X-ray diffraction and aerodynamic levitation to investigate the structures of liquid FeO_*x*_ with 1 < *x* < 1.5, which corresponds to the range 0.5 < *Z*_O_ < 0.6, in the range of a supercooled liquid at 1573 K up to the stable melt at 1973 K. They found that the local Fe–O coordination number is in the range of 4–5, which is very different from the (solid phase) crystalline phases.

In this work, the Fe–O and O–Fe coordination numbers are obtained from the MD simulations. Instead of indicating the oxidation degree by *Z*_O_, the same notation as that of Shi et al.^38^ will be used, defined as
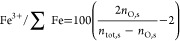
4with *n*_O,s_ being the number of oxygen atoms and *n*_tot,s_ the total number of atoms in the complete domain and where Fe^3+^/∑Fe = 0% for *Z*_O_ = 0.5 and Fe^3+^/∑Fe = 100% for *Z*_O_ = 0.6.

**Figure 9:**
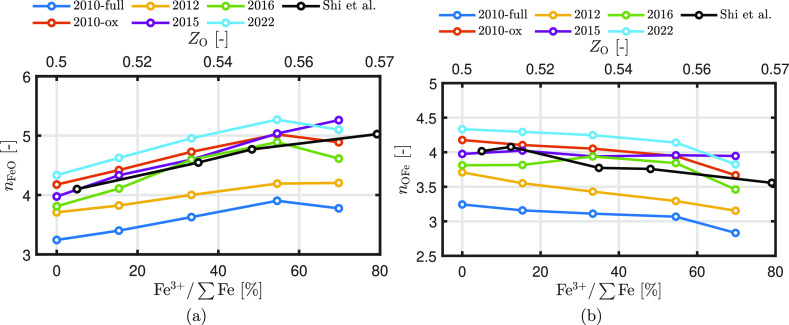
(a) Fe–O and (b) O–Fe coordination numbers in liquid iron oxide at 2000 K as a function of the degree of oxidation. The experimental data of ref (38) are added as references.

Figure 9 shows the Fe–O and O–Fe coordination numbers in liquid iron oxide at 2000 K as a function of the oxidation degree. The black solid line indicates the experimental data of Shi et al.^38^ The first minimum of the Fe–O RDF is used as the cutoff distance to determine the coordination numbers. It can be seen that the liquid structures in this highly oxidized regime are well captured by ReaxFF2010-ox, ReaxFF2015, and ReaxFF2016, while ReaxFF2010-full and ReaxFF2012 systematically underestimate and ReaxFF2022 systematically overestimates the coordination numbers. Since ReaxFF2010-ox, ReaxFF2015, and ReaxFF2016 predict coordination numbers within 5%, we categorize them as “good”. ReaxFF2012 and ReaxFF2022 predict the values within 10% (categorization: “moderate”) and ReaxFF2010-full within 20% (categorization: “bad”).

It can be remarked that for ReaxFF2015, even though it was shown before that the RDF of liquid FeO implies a solid-phase structure, the coordination number is well below that of a crystalline FeO structure^38^ (*n*_FeO_ = 6) and captures the coordination of the molten states quite accurately. This observation can be explained by the fact that ReaxFF2015 already does not accurately predict the coordination number of the solid-phase properties. Specifically, it predicts a coordination number of approximately 4.5 for solid FeO at 300 K rather than 6.

### 3.4 Transport Properties

Transport properties of liquid iron oxide are important to model the oxidation of an iron droplet, especially in cases where the gaseous O_2_ concentration is sufficiently high so that external O_2_ diffusion and surface chemisorption are not the rate-limiting factors.^14^ To model the internal structure of a liquid iron oxide droplet during combustion, the diffusion coefficients of Fe and O are needed to model the internal diffusion. Furthermore, viscosity is needed to model the possible convective flow occurring in a liquid droplet. Therefore, the different ReaxFF parameter sets are examined in determining these transport properties as a function of the degree of oxidation at 2000 K.

#### 3.4.1 Diffusion Coefficients

**Figure 10:**
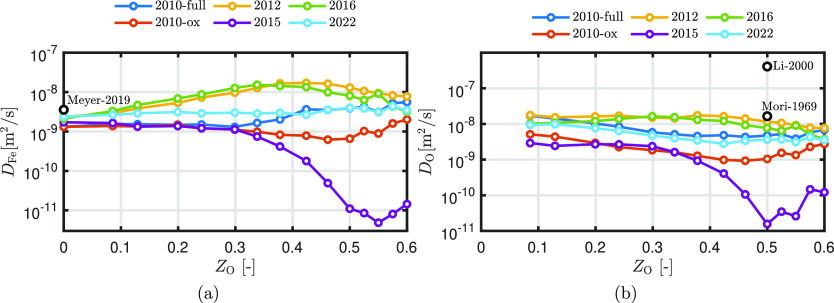
Diffusion coefficients of (a) Fe and (b) O as a function of the degree of oxidation. The experimental self-diffusion coefficient of Fe at 1940 K (Black circle) is added as a reference.^49^

A common method of finding the diffusion coefficient through MD simulation is by calculating the slope of the mean-square displacement of the atoms as
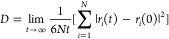
5where *N* is the number of atoms, and *r*_*i*_(0) and *r*_*i*_(*t*) are the positions of the atom *i* in the initial state and after time *t*. Figure 10 shows the diffusion coefficients of Fe and O as a function of the degree of oxidation. The experimental self-diffusion coefficient of Fe at 1940 K^49^ and the diffusivity of O according to Mori and Suziki^51^ and Li et al.^50^ at, respectively, 1823 and 1773 K are added as references. For the diffusion of oxygen in liquid iron oxide, the limited reported values^50,51,76,77^ differ by 2 orders of magnitude. The values of Grieveson and Turkdogan^76^ and Mori and Suziki^51^ are generally in the order of  and , while the experimental determined values of Sayadyaghoubi et al.^77^ and Li et al.^50^ are in the order of . The latter two authors state that this difference in magnitude arises from neglecting the limiting mechanism of surface chemical reaction between iron oxide and the oxidizing gas in the work of Grieveson and Turkdogan^76^ and Mori and Suziki.^51^

All of the ReaxFF parameter sets seem to underestimate the self-diffusion coefficient of Fe with a factor of approximately 2 but are in the same order of magnitude. The MD-obtained diffusivity of oxygen is 1 order smaller compared to the experimentally derived value of Li et al.,^50^ which are expected to be the most accurate, but are similar to the values of Mori and Suziki.^51^ The faster diffusivity of O compared to Fe can be related to the smaller atomic radius of O in Fe (*r*_FeO_) compared to that of Fe in Fe (*r*_FeFe_),^23^ defined as the position of the first peak of the RDF. The atomic radius *r*_FeO_ varies between 1.8 and 2 Å, while *r*_FeFe_ varies between 3 and 3.1 Å, which implies that O is more mobile than Fe.

ReaxFF2010-full, ReaxFF2010-ox, and ReaxFF2022 predict similar trends for the diffusion coefficients of Fe and O, but they differ in magnitude. These predicted diffusion coefficients of O decrease with an increasing oxidation degree. The trend for ReaxFF2012 and ReaxFF2016 differs from the others, where *D*_Fe_ increases significantly as a function of *Z*_O_ and is around 3 times compared to the other ReaxFF parameter sets at *Z*_O_ = 0.3. Note that for ReaxFF2016, phase separation is observed, implying that the local degree of oxidation where oxygen diffusion occurs is greater than the overall oxidation degree of the system. The trend of ReaxFF2015 deviates significantly from the others, showing diffusion coefficients that are around 2 orders of magnitude lower than the others. This deviation is consistent with the fact that ReaxFF2015 fails to capture the liquid-phase structure at high oxidation degrees, as shown in Section 3.2.1. The experimental results of Li et al.^50^ and Mori and Suziki^51^ show a decrease of oxygen diffusion with an increasing oxidation degree, which is generally in line with our results. For studies related to Earth-core conditions, it is found that the diffusivity of Fe increases with an increasing oxygen concentration although the dependence of oxygen diffusivity on *Z*_O_ is unclear.^25^

The comparison of the NEB calculations for diffusion with DFT shows that ReaxFF2022 reasonably predicts the diffusion of the oxygen atom in iron. Furthermore, ReaxFF2010-ox predicts the same MEP, but it overestimates the energy barrier. Therefore, it is most likely that these two ReaxFF parameter sets are suitable to predict the diffusion coefficients in the Fe–O system. However, due to the lack of experimental data, only ReaxFF2015 is categorized as “bad”, while for the others, no categorization is done.

#### 3.4.2 Viscosity

There are multiple methods in molecular dynamics to determine viscosity. These methods can be distinguished as nonequilibrium methods and equilibrium methods. Nonequilibrium methods for viscosity determination in MD simulations involve imposing external forces or gradients to induce flow and measuring the resulting shear stress. Equilibrium methods, on the other hand, rely on fluctuation analysis to calculate the viscosity by examining the temporal and spatial correlations of the velocities of the system. In this work, the Green–Kubo approach is used as an equilibrium method. In the Green–Kubo approach, the shear viscosity is calculated from the integral over time of the pressure tensor autocorrelation function^78^
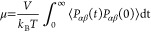
6where *V* is the system volume, *k*_B_ is the Boltzmann constant, *T* is temperature, *P*_αβ_ denotes the element αβ of the pressure tensor, and the angle bracket indicates the ensemble average.

**Figure 11:**
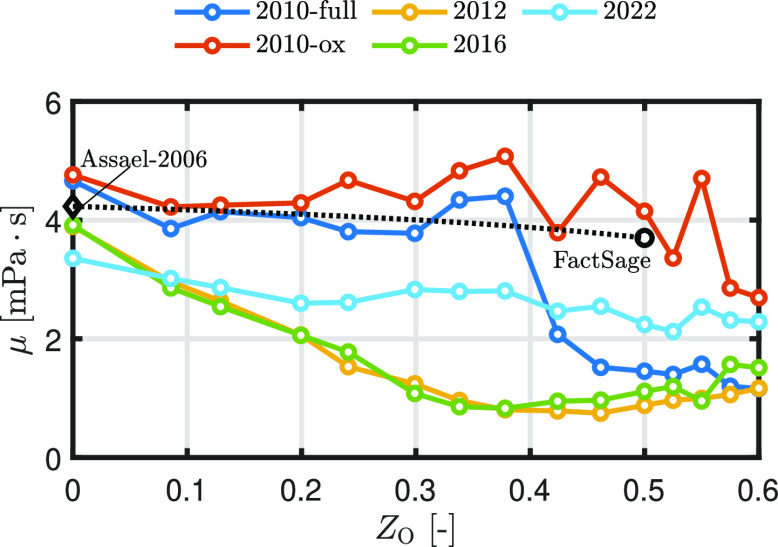
Trends in the viscosity of liquid iron oxide systems as a function of the oxidation degree. The surface temperature is equal to 2000 K. The experimentally^52^ obtained values (black diamonds) and the viscosity calculated with FactSage (black circle) are shown as references. The black dotted line is a linear interpolation of the experimental values based on the molar fraction of liquid Fe and liquid FeO. The values of ReaxFF2015 are not shown; since this ReaxFF does not predict the liquid phase properly, the obtained viscosity values are not reliable.

Figure 11 shows the viscosity of the liquid Fe–O systems as a function of the oxidation degree at 2000 K. Note that in the case of phase separation, this equals the average viscosity of the complete system. The experimentally obtained viscosity of pure liquid iron is depicted with the black diamond,^52^ while the viscosity of liquid FeO calculated with FactSage is shown with the black circle. The black dotted line is a linear interpolation of the experimental values based on the molar fraction of liquid Fe and liquid FeO. The values of ReaxFF2015 are not shown; since this ReaxFF does not predict the liquid phase properly, the obtained viscosity values are not reliable. All the ReaxFFs reasonably predict the viscosity of liquid Fe. However, they start to deviate with the increasing oxidation degree, although they remain within the same order of magnitude. ReaxFF2010-ox gives the best prediction of the viscosity of liquid FeO, while ReaxFF2016 shows the largest deviation with a factor of approximately 4. In general, the diffusivity and viscosity are related via the Stokes–Einstein (S–E) relation as^25,79^
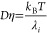
7where λ_*i*_ is a factor related to the radius of the diffusing unit of species *i* in a liquid. According to the S–E relation, an increasing diffusion coefficient results in a decreasing viscosity, which is also observed in the MD simulation results. Under Earth-core conditions, it is also found that the viscosity decreases with an increasing oxygen concentration.^25,71^

It is argued that the trends in transport properties predicted by ReaxFF2010-ox and ReaxFF2022 are the most probable following an evaluation of the NEB findings and a comparison between the MD simulation results and the literature and thermodynamic values for viscosity. Since the trend of ReaxFF2010-ox is close to the literature values, we categorize it as “good”. ReaxFF2022 predicts the same trend as ReaxFF2010-ox, but it does differ by at least 40% with respect to the literature values. ReaxFF2010-full shows a similar trend as ReaxFF2010-full until *Z*_O_ = 0.4 but then drops and underestimates the viscosity of liquid FeO with 60%. Therefore, these ReaxFF parameter sets are categorized as “moderate”, and the remaining ReaxFF parameter sets are categorized as “bad”.

### 3.5 Mass and Thermal Accommodation Coefficients

For iron particle combustion, the MAC and TAC are important parameters which influence the particle temperature during combustion.^14^ Since the heat and mass transfer in the free-molecular regime are dependent on the TAC and MAC, it is important to derive reliable values for the TAC and MAC.

The MAC or absorption coefficient is defined as the fraction of incoming oxygen molecules that upon collision with the iron surface are absorbed (accommodated) rather than being reflected. The MAC is defined as
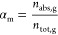
8with *n*_abs,g_ being the number of absorbed gas molecules and *n*_tot,g_ the total number of gas molecules colliding the surface. The oxygen molecules that do not stick to the surface during Fe_*x*_O_*y*_–O_2_ interactions still contribute to the TAC. The TAC describes the average energy transfer when gas molecules scatter from the surface and is defined as
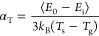
9with ⟨·⟩ denoting an ensemble average, *E*_0_ the total energy of the scattered molecule, and *E*_i_ the energy of the incident molecule. The denominator represents the maximum energy that could be transferred from the surface to the gas molecule, with *T*_s_ the surface temperature and *T*_g_ the gas temperature.

The MAC and TAC for iron with oxygen are investigated for different initial oxidation stages, ranging from *Z*_O_ = 0 to *Z*_O_ = 0.57, and *T*_s_ = 2000 K. A gas temperature of *T*_g_ = 300 K is used. The distance between the two O atoms of the incident oxygen molecule is used as a metric for the MAC. If the interatomic distance becomes larger than 1.2 times the initial bond length, the incident oxygen molecule is considered as dissociated and chemically absorbed into the iron(-oxide) surface. Since it has been demonstrated that ReaxFF2015 is unable to predict the characteristics of liquid iron oxide, this ReaxFF is not used to calculate the TAC and MAC.

**Figure 12:**
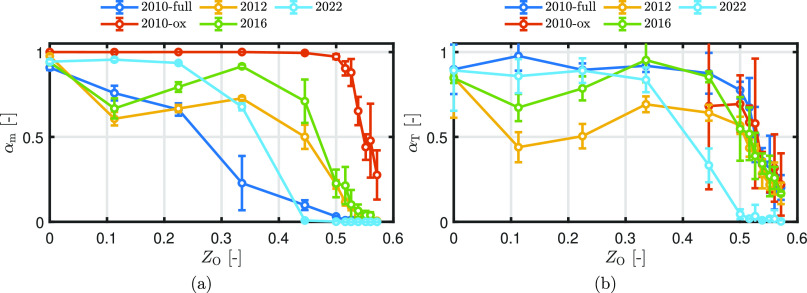
(a) MAC and (b) TAC between Fe_*y*_O_*x*_ and O_2_ as a function of initial oxidation stage at a surface temperature *T*_s_ = 2000 K and a gas temperature *T*_g_ = 300 K.

Figure 12 shows the MAC and TAC of oxygen as a function of the initial oxidation stage for the different ReaxFF parameter sets. Note that the results of ReaxFF2010-full are used in the work of Thijs et al.^14^ The MAC differs significantly among the different ReaxFF parameter sets. ReaxFF2010-full shows a nearly linear decrease with an increasing oxidation degree in the region 0 < *Z*_O_ < 0.5, while the MAC of ReaxFF2010-ox remains close to unity in this region. When both MACs are applied to a single iron particle combustion model,^14^ the result for ReaxFF2010-ox shows a fully external diffusion-limited combustion mode, while for ReaxFF2010-full, the particle burns in a regime in between external diffusion-limited and kinetic- (or chemical-) absorption-limited regime. In the region *Z*_O_ > 0.5, all of the ReaxFFs show a decrease in the MAC, which implies that the oxidation becomes significantly more difficult as the oxidation degree further increases. However, the results from the ReaxFF parameter sets differ by nearly 2 orders of magnitude, with ReaxFF2022 predicting zero values.

As shown with the NEB simulations, ReaxFF2010-ox overestimates the change in potential energy for the adsorption case. The large potential energy change predicted by ReaxFF2010-ox indicates a strong attraction between the Fe surface and the incoming oxygen molecule, resulting in an MAC close to unity. Therefore, the MAC predicted by ReaxFF2010-ox is likely overestimated.

**Figure 13:**
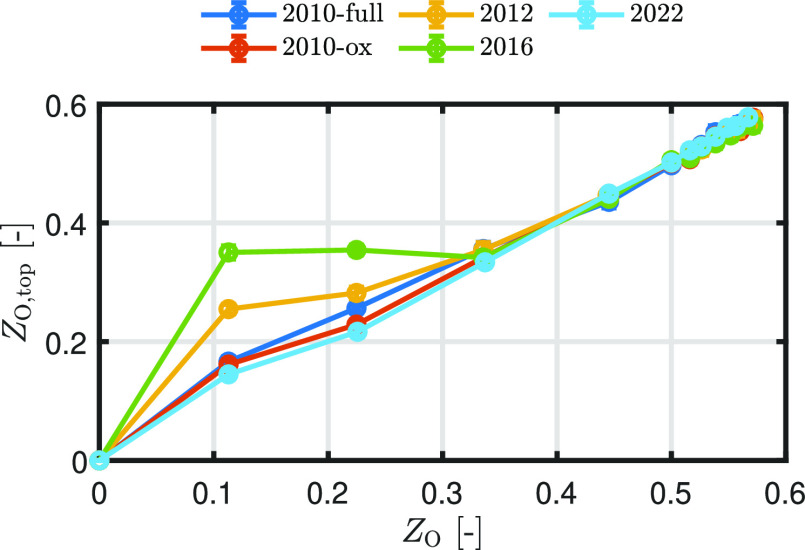
*Z*_O_ determined over the entire domain compared to *Z*_O_ evaluated within the upper 10 Å of the surface, denoted by *Z*_O,top_. The data are averaged over 50 timesteps, with error bars representing the standard deviation.

Note that due to the immiscibility predicted for ReaxFF2016, the total *Z*_O_ of the domain varies with respect to the *Z*_O_ value of the surface, which is exposed to the incoming oxygen molecule. Figure 13 shows *Z*_O_ determined over the entire domain compared to *Z*_O_ evaluated within the upper 10 Å of the surface, denoted by *Z*_O,top_. The data are averaged over 50 timesteps, with error bars representing the standard deviation. Especially for ReaxFF2016, and slightly for ReaxFF2012, *Z*_O,top_ differs from the overall *Z*_O_ due to phase separation. Therefore, the incoming oxygen molecule experiences a locally higher *Z*_O_ than the averaged *Z*_O_ over the entire domain. This explains why the MAC for ReaxFF2012 and ReaxFF2016 roughly plateaus in the region 0 < *Z*_O_ < 0.4.

On the other hand, the ReaxFF parameter sets predict similar trends for the TAC. When the oxidation degree of the system is low, the TAC remains close to unity, while ReaxFF2012 predicts a TAC which is around 0.5 in the range 0 < *Z*_O_ < 0.45. Then, the TAC decreases sharply if *Z*_O_ > 0.5. This drop can be explained by means of the residence time of the molecules on the surface. With an increasing *Z*_O_, incoming oxygen molecules are repelled by the surface, resulting in a less residence time on the surface. Therefore, the oxygen molecule has less time to equilibrate with the surface, resulting in a lower TAC.^80^ Note that in the region 0 < *Z*_O_ < 0.45, there is no TAC reported for ReaxFF2010-ox since the MAC is equal to unity.

Unfortunately, for both the TAC and MAC between Fe_*x*_O_*y*_ and O_2_, no experimental data are available to validate the MD-derived data. However, the mismatch between the NEB simulations and DFT calculations for the adsorption case suggests that ReaxFF2010-ox overpredicts the MAC.

#### 3.5.1 Assessment of the MAC and TAC Using the Single Iron Particle Combustion Model

**Figure 14:**
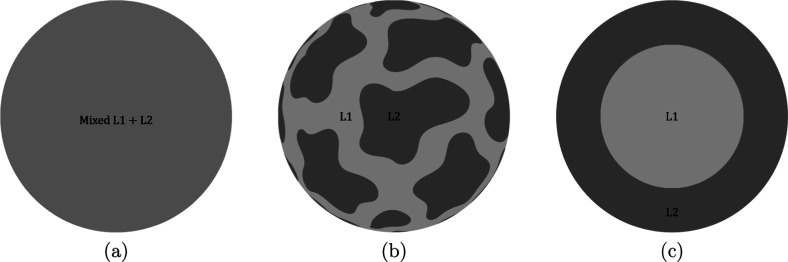
Schematic of different hypothetical particle morphologies of a burning liquid iron oxide particle. (a) Homogeneous mixed particle, (b) unmixed (cow-like pattern) particle, and (c) core–shell particle.

To assess the impact of the MAC and TAC obtained with the different ReaxFF parameter sets, the single iron particle model, as described by Thijs et al.,^14^ is used. In the study of Thijs et al.,^14^ it was assumed that the iron oxide particle consists of a homogeneous mixture. However, as previously discussed, the equilibrium phase diagram^37^ describes that liquid iron (L1) and liquid iron oxide (L2) do not mix in the region between 0.013 < *Z*_O_ < 0.506 at 2000 K. Furthermore, the experimental observations of Muller et al.^20^ seem to indicate a complex oxidation process in the liquid, for instance, with L1 being also present at the particle surface and, under certain circumstances, miscibility between the phases L1 and L2. Here, the impact of the MAC and TAC obtained with the different reactive force fields is tested under three different hypothetical particle internal structures, as illustrated in Figure 14. The particle can either be a miscible homogeneous mixture, a randomly scattered immiscible structure (unmixed), or an immiscible core–shell structure. Here, the unmixed model is drawn with a cow-like pattern, but it can have different kinds of unmixed structures, e.g., L1 on one side and L2 on the other side.^81^ Note that in the case of an unmixed structure, parts with L1 oxidize faster than parts with L2, resulting in a strong diffusion of gaseous oxygen toward the L1 region and an asymmetric flow pattern around the particle.

Table 3 states how MAC changes as a function of oxidation degree for the three different hypothetical particle morphologies, as shown in Figure 14. It is assumed that for L1, *Z*_O_ = 0 and for L2, *Z*_O_ = 0.5. Note that in the equilibrium phase diagram the boundaries of the miscibility gap converges with increasing temperature.^37^

**Table 3:** α_m_ Relations Used for the Different Hypothetical Particle Morphologies

particle model	α_m_ relation
homogeneous mixed	α_m_ = *f*(*Z*_O_)
unmixed	
core–shell	α_m_ = *f*[max(*Z*_O_, L2)]

**Figure 15:**
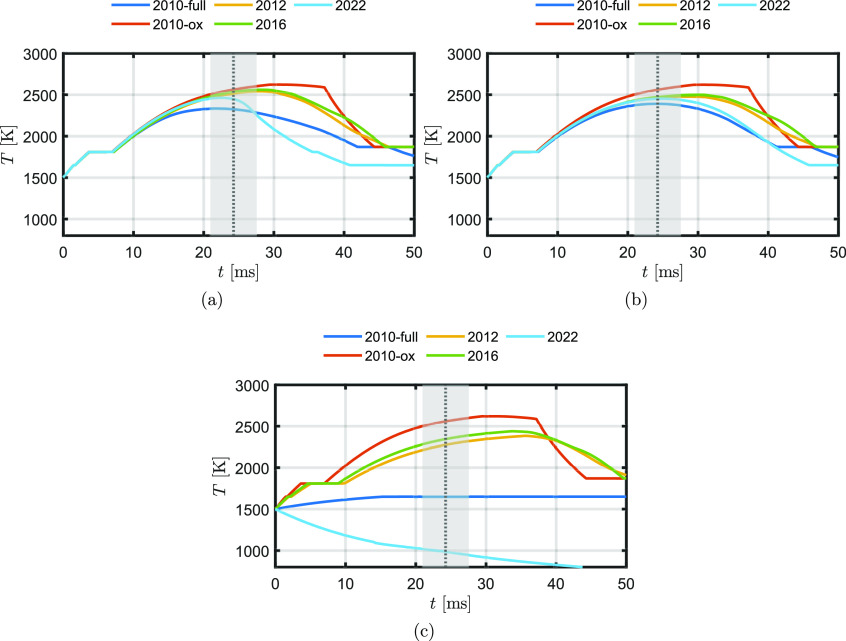
Effect of different ReaxFF parameter sets and hypothetical particle morphologies on the temperature vs time curve of a 54 μm particle burning in a gas of *T*_g_ = 300 K with *X*_O2_ = 0.21. (a) Homogeneous mixed particle, (b) unmixed particle, and (c) core–shell particle. The dotted line is the time to maximum temperature *t*_max_, as reported by Ning et al.^2^ The gray area represents the standard deviation at *t*_max_.

Figure 15 shows the impact of the different ReaxFF parameter sets and particle morphologies on the temperature curve of a 54 μm particle burning in a gas of *T*_g_ = 300 K with *X*_O2_ = 0.21. The dotted line is the time to maximum temperature *t*_max_, as reported by Ning et al.^2^ The gray area represents the standard deviation at *t*_max_. An initial particle temperature of *T*_p,0_ = 1500 K is considered.

For the homogeneous mixture, the burn time shows a good match when using ReaxFF2010-full and ReaxFF2022, while the three other ReaxFFs overpredict the burn time. While ReaxFF2022 shows good agreement with the experimental burn time, the final oxidation degree is not in line with the work of Choisez et al.^82^ They showed that oxide particles produced by iron combustion in air consist of mainly magnetite (Fe_3_O_4_) and a small amount of hematite (Fe_2_O_3_), thus indicating an overall oxidation degree of *Z*_O_ > 0.5. Since the MAC for ReaxFF2022 is equal to zero in the region *Z*_O_ > 0.5, the particle does not oxidize further than stoichiometric FeO and therefore does not form magnetite or hematite. For ReaxFF2010-ox, the temperature curve does not show a smooth transition at the maximum temperature compared to the other temperature curves, which is a result of the high MAC in the region *Z*_O_ > 0.5. For ReaxFF2010-full, ReaxFF2012, and ReaxFF2016, the particle peak temperature is at the position where the rate of heat loss exceeds the rate of heat release while the particle is not yet fully oxidized. For ReaxFF2010-ox, the MAC in the region *Z*_O_ > 0.5 is relatively high. Therefore, the maximum temperature is attained at the point where the particle is fully oxidized: the available iron is completely oxidized, and therefore the heat release rate immediately drops to zero. If *t*_max_ matches with the experimental value, we categorize it as “good”. If *t*_max_ is close to the standard deviation, we categorize it as “moderate” and otherwise as “bad”. It is important to note that based on this classification, ReaxFF2012 is categorized as “moderate” and “bad,” despite having been classified as “good” in the case of MEP adsorption. This discrepancy indicates that the results from the adsorption NEB simulations may not be suitable for assessing the MAC. Alternatively, it suggests that the single iron particle model lacks the required level of accuracy.

Overall, the homogeneous mixture and the unmixed model do not differ much from each other. When using a core–shell structure, the cases with ReaxFF2010-full and ReaxFF2022 do not burn sufficiently, since the MAC is too low or equal to zero in the region *Z*_O_ > 0.5. With the other ReaxFF parameter sets, the time to maximum temperature significantly differs from the experimental results. Based on these results, it is unlikely that the particle in the experiments of Ning et al.^2^ burns in a core–shell mixture. Experimental research is needed to investigate the particle morphology achieved during iron particle combustion.

### 3.6 Summary of Findings

The MD-derived properties corresponding to various ReaxFF parameter sets are compared to five critical parameters. In Table 4, an assessment is provided categorizing the performance of different ReaxFF parameter sets as either good, moderate, or bad, in relation to the available benchmark values. It is important to note that the categorizations, i.e., “bad”, “moderate”, and “good”, are used to assess the relative performance among different ReaxFF parameter sets in predicting certain properties of the Fe–O system. For a detailed explanation of the rationale behind these categorizations, we direct the reader to the specific section corresponding to each property. It is evident that notable discrepancies exist among the MD simulation outcomes achieved with different ReaxFF parameter sets. Since the liquid iron oxide phase is beyond the training set targets for any of these force fields, the MD simulation results widely scatter. Based on the available benchmark, it can be concluded that there is no optimal ReaxFF available for a liquid Fe–O system. The results emphasize the necessity for an improved ReaxFF model to accurately represent the properties of a liquid Fe–O system. This table can guide the choice of the appropriate ReaxFF for applications in which the importance of a specific parameter (or a subset of them) is dominant.

**Table 4:** Categorization of the Performance of Different ReaxFF Parameter Sets as Either Good, Moderate, or Bad in Relation to the Available Benchmark Values^a^

	properties	ReaxFF2010-full	ReaxFF2010-ox	ReaxFF2012	ReaxFF2015	ReaxFF2016	ReaxFF2022
1a.	MEP: adsorption	moderate	bad	good	moderate	moderate	moderate
1b.	MEP: diffusion	bad	good	bad	bad	bad	good
2a.	density	moderate	moderate	moderate	bad	moderate	moderate
3a.	enthalpy of mixing	bad	bad	moderate	good	good	bad
3c.	immiscible phase: L2 composition	UtP	UtP	UtP	bad	bad	UtP
3d.	miscibility phase: coordination numbers	bad	good	moderate	good	good	moderate
4a.	diffusion coefficients	LoB	LoB	LoB	bad	LoB	LoB
4b.	viscosity	moderate	good	bad	bad	bad	moderate
5a.	burn time prediction						
	homogeneous	good	bad	moderate	N.A	moderate	moderate
	unmixed	good	bad	bad	N.A	bad	moderate
	core–shell	bad	bad	bad	N.A	bad	bad

a In the case of the lack of benchmark data (LoB), or if the ReaxFF is unable to predict the property (UtP), or the ReaxFF parameter set is not used (N.A.) for the specific property, no categorization is performed.

## 4 Conclusions

Reactive molecular dynamics simulation is a useful tool for determining several physical properties. However, prudence must be practiced in the selection of the reactive force field. This work investigates how six different ReaxFF parameter sets influence the MD-derived properties of liquid Fe–O systems. Since the liquid iron oxide phase is beyond the training set targets for any of these force fields, the MD simulation results widely scatter. Based on NEB simulations, ReaxFF2012 seems to be the best candidate to predict the adsorption of O on the Fe surface, while ReaxFF2010-ox and ReaxFF2022 predict the MEP for diffusion the most accurate. ReaxFF2015 and ReaxFF2016 are the only reactive force fields that predict the miscibility gap between liquid- and liquid iron oxide, but the phase compositions do not match with the equilibrium Fe–O phase diagram. Furthermore, ReaxFF2015 fails to predict the liquid iron oxide structure but instead shows properties that belong to a solid phase. Even though ReaxFF2016 seems to predict the density, immiscibility, and coordination number of the liquid iron oxide quite accurately, it fails to predict the viscosity of liquid FeO and therefore probably also the diffusion coefficients. All the ReaxFF parameter sets predict different trends for the MACs as a function of the oxidation degree, which will have a significant impact on modeling the combustion of single iron particles.

Significant discrepancies among the MD simulation results using different ReaxFF parameter sets have been observed. Based on the available experimental data and equilibrium calculation results, an improved ReaxFF is required to better capture the properties of a liquid Fe–O system.
